# A computational method for identification of vaccine targets from protein regions of conserved human leukocyte antigen binding

**DOI:** 10.1186/1755-8794-8-S4-S1

**Published:** 2015-12-09

**Authors:** Lars R Olsen, Christian Simon, Ulrich J Kudahl, Frederik O Bagger, Ole Winther, Ellis L Reinherz, Guang L Zhang, Vladimir Brusic

**Affiliations:** 1 Cancer Vaccine Center, Dana-Farber Cancer Institute, Boston, MA, USA; 2 Bioinformatics Centre, Department of Biology, University of Copenhagen, Copenhagen, Denmark; 3 Center for Biological Sequence Analysis, Department of Systems Biology, Technical University of Denmark, Lyngby, Denmark; 4 Biotech Research and Innovation Center (BRIC), University of Copenhagen, Copenhagen, Denmark; 5 Disease Systems Biology, Novo Nordisk Center for Protein Research, University of Copenhagen, Copenhagen, Denmark; 6 The Finsen Laboratory, Rigshospitalet, Faculty of Health Sciences, University of Copenhagen, Copenhagen, Denmark; 7 Laboratory of Immunobiology, Dana-Farber Cancer Institute, Boston, MA, USA; 8 Department of Medicine, Harvard Medical School, Boston, MA, USA; 9 Department of Computer Science, Metropolitan College, Boston University, Boston, MA, USA; 10 School of Medicine and Bioinformatics Center, Nazarbayev University, Astana, Kazakhstan

**Keywords:** bioinformatics, T cell immunity, epitope prediction, conservation analysis, cross-reactivity

## Abstract

**Background:**

Computational methods for T cell-based vaccine target discovery focus on selection of highly conserved peptides identified across pathogen variants, followed by prediction of their binding of human leukocyte antigen molecules. However, experimental studies have shown that T cells often target diverse regions in highly variable viral pathogens and this diversity may need to be addressed through redefinition of suitable peptide targets.

**Methods:**

We have developed a method for antigen assessment and target selection for polyvalent vaccines, with which we identified immune epitopes from variable regions, where all variants bind HLA. These regions, although variable, can thus be considered stable in terms of HLA binding and represent valuable vaccine targets.

**Results:**

We applied this method to predict CD8^+ ^T-cell targets in influenza A H7N9 hemagglutinin and significantly increased the number of potential vaccine targets compared to the number of targets discovered using the traditional approach where low-frequency peptides are excluded.

**Conclusions:**

We developed a webserver with an intuitive visualization scheme for summarizing the T cell-based antigenic potential of any given protein or proteome using human leukocyte antigen binding predictions and made a web-accessible software implementation freely available at http://met-hilab.cbs.dtu.dk/blockcons/.

## Background

Along with sanitation, vaccines are the most effective and economic public health tools for control of infectious disease [1]. However, vaccine development faces a number of challenges, such as overcoming the limited effectiveness of a number of vaccines, the need for frequent vaccine reformulation, as well as a complete lack of vaccines for some diseases. A central goal of vaccination is to generate long lasting and broadly protective immunity against target pathogens, but this goal is hampered by the variability of both the target pathogens and the human immune system [2]. Current practical solutions to the problem include polyvalent vaccines such as those being developed for dengue virus [3] or seasonal vaccine reformulation against influenza [4].

The majority of traditional vaccines provide protection through neutralizing antibodies and T cells alone rarely offer protection and prevention of diseases. However, they participate in reduction, control, and clearance of intracellular pathogens and have been linked with protective immunity against a number of viral pathogens [5-8]. The biggest success of immunological bioinformatics is the development of algorithms for prediction of peptide binding affinity to the human leukocyte antigen (HLA) - one of the rate limiting steps in T cell-based immune response [9]. Although current forms of these algorithms are highly accurate [10-12], the output alone is not enough to inform the selection of epitopes for therapeutic applications. In the conceptual framework for reverse vaccinology, Rino Rappouli described *in silico *predictions of immune epitopes from biological sequence data as a "naïve approach" when compared with experimental elucidation immunogenic peptides. Many parameters of a good vaccine target conferring efficient, lasting immunity, still remain to be considered after prediction of HLA binding: multiple rate-limiting steps of peptide pre-processing, confirming *in vivo *expression, considering dynamics of expression in different developmental stages and cellular environments, presence of epitope across pathogen population, response across host population, epitope stability over time, and others [13]. Here, we address the issue of variability by modifying the antigen selection step with a computational method for selecting multiple T cell targets from functionally homologous protein regions.

Traditionally, vaccine targets are selected from conserved regions in the genome of the pathogen in question, with the aim of conferring broad and lasting immunity. The first step is a variability analysis performed by calculating the frequency of nucleotides or amino acids on each position in a multiple sequence alignment (MSA) of homologous genes or proteins [14]. Regions, in which several consecutive residues show high conservation (typically >90% conservation is chosen as the threshold), are then further analyzed for immunogenic potential either by computational predictions, experimental testing, or a combination thereof. This systematic exclusion of low frequency variants when using traditional approaches [15-19] represents a major limiting factor, since immunogenic potential does not always correlate with the frequency in the viral population - both rare and common peptides can be immunogenic and valuable in vaccine constructs aiming for broad coverage [20].

Since the human immune system's evolution occurs on a significantly longer time scale than rapidly mutating pathogens [21], high selective pressure causes them to alter expression of some immunogenic antigens faster than the immune system can evolve to keep up with the changes [22]. The HLA binding affinity of a peptide relative to its frequency in a viral or malignant cell population is known as its targeting efficiency (TE). It has been shown that the TE of peptides varies in different organisms, and in some highly variable viruses it tends to be low [20]. Regions of high TE comprise peptides that are highly conserved, most likely owing to the protein's functional importance limiting the capacity of a pathogen to alter the protein while maintaining its fitness [23]. Regions of low TE comprise one or more peptides, potentially all of high HLA binding affinity, but each of them will have a low frequency in the pathogen population. For rapidly mutating viruses, such as RNA viruses [24] the selective pressure exerted on HLA-binding peptides, means that host immunity will often, and in some cases preferentially, target low frequency epitopes [20].

### Selecting vaccine targets from protein regions with conserved HLA binding

**Figure 1:**
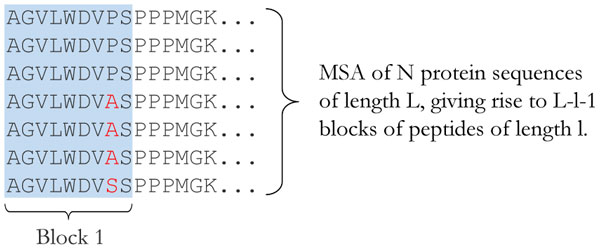
**Extraction of blocks from MSA**. Subdivision of an MSA into blocks of peptides, *l *amino acids in length. In this example, *l = 9*. Block 1 is highlighted in blue. Moving the sliding window to the right in the MSA in increments of one position will give all blocks blocks in the MSA.

We propose a novel method and visualization scheme for assessing the stability of protein regions for T cell target discovery, which takes the evolutionary relationship between HLA and pathogen epitopes into consideration. This method is based on analyzing columns of suitably sized sliding windows (from here on termed "blocks") from the rows of sequences in an MSA (Figure 1). An MSA of homologous protein sequences can be performed using a number of algorithms [25], and blocks of peptides of a given size (usually 8-11 amino acids long for HLA class I restricted epitopes and 13-25 amino acids long for HLA class II restricted T-cell epitopes) are extracted from each position in the alignment. The number of peptides in each block indicates the diversity of the block, for which Shannon entropy and consensus frequency can be calculated as informative metrics [26].

In order to identify potential T cell targets, HLA binding affinities are predicted for all peptides in all blocks. Because blocks are extracted from aligned regions of homologous proteins, it is likely that the peptides within a given block display high sequence homology and the majority show similar HLA binding properties even when sequence variations exist. Similarly, the regions surrounding a block will be of high homology, thus increasing the likelihood that peptides from the same block will be processed and presented on the surface of target cells in a similar fashion [27].

Blocks of one or more peptides that are all predicted to bind to the same HLA alleles with similar affinity are potentially valuable targets for polyvalent vaccine designs. This allows for simultaneous immunization with several epitopes - a necessary tactic against highly mutating viruses in which mutations introducing drug resistance can occur within a single day [28,29]. We previously used a rudimentary version of the block conservation analysis for vaccine target discovery in dengue virus (DENV) [30] and reported a 10-fold larger number of potential CD8^+ ^vaccine target candidates as compared to an earlier benchmark study of DENV vaccine target candidates [31]. We here formalize the approach and present a software implementation. To further demonstrate the utility of block conservation, we performed an analysis of HLA class I epitopes in influenza A H7N9 hemagglutinin (HA). The software is integrated into a freely available web service at http://met-hilab.cbs.dtu.dk/blockcons/.

## Methods

### Multiple sequence alignments

MSAs were performed using MAFFT [32]. When aligning highly variable protein sequences, such as proteins from influenza virus, MSAs will invariably contain a high proportion of gaps. Gaps, typically denoted by a dash "-", are artifacts of the MSA algorithms and distort the analysis of peptides in blocks derived from MSAs. We developed and applied an algorithm that addresses this by removing gaps and extending the length of the resulting shortened peptides to match the block length. Extensions are made either upstream or downstream, depending on which direction yields the best short read alignment with the rest of the block.

### Block conservation

Variability metrics are based on information content calculated as Shannon entropy [26] and conservation is defined as the frequency of the predominant peptide. In the block conservation analysis we calculate the information content and frequency of all peptides in an MSA of *N *homologous proteins, as previously described [30]. Briefly, the peptides at starting position *x *are here collectively referred to as a "block", *B_x_*. The peptides in each block have user-defined length, *l*, and in each given block, a number, *W_x_*, of unique peptides exist. Starting from the first position *x = 1 *and increasing *x *in increments of one across the entire MSA of length *L *results in *L-l+1 *blocks. The block conservation is assessed using the minimum percentage, *y_x_*, of a block at starting position *x *that must be covered by a subset of peptides, *S_w_*, for a block to be considered conserved. In our analyses of the influenza A HA protein for class I binders, the following parameters were used: *l *= 9, *y_x _*= 99%.

### Data

The data used to exemplify the application of block conservation was extracted from FluKB [33]. We used 148 full sequences from influenza A H7N9 for this analysis.

### T-cell epitope prediction

HLA class I binding affinities of peptides were predicted using NetMHC 3.4 [34]. This method was shown to offer good accuracy relative to other available methods [10,11]. The default thresholds for binding level affinity (IC_50 _< 500 nM for weak binders and IC_50 _< 50 nM for strong binders) were used for binding prediction. Predictions were performed for the following class I alleles: A*01:01, A*02:01, A*03:01, A*11:01, A*24:02, B*07:02, B*08:01, B*15:01. These alleles were chosen since prediction algorithms have proven most accurate for these alleles [12]. For prediction of binding to all known HLA class I alleles, NetMHCpan 2.4 [35] can be used (Additional file 1). This algorithm enables prediction of peptide binding to 886 HLA-A alleles, 1412 HLA-B alleles, and 617 HLA-C alleles, as opposed to NetMHC 3.4, which only enables prediction for 34 HLA-A alleles, 33 HLA-A alleles, and 10 HLA-C alleles.

## Results and discussion

### Visualizing block conservation analysis results

Given the large quantity of outputs from the block conservation analysis and HLA binding predictions, we developed visualizations to provide a convenient way of summarizing results. The conservation of blocks is visualized using bar plots of the minimum number of peptides required to fulfill the user-defined coverage threshold, *y_x_*, (Y axis) for each starting position in an MSA of the protein or proteome in question (X axis). The predicted binding affinities of peptide blocks to the user-specified HLA-I or HLA-II alleles are visualized using a heat map displayed below the conservation graph, where each column in the heat map corresponds to the binding affinity of the given position in the MSA, and each row corresponds to an HLA allele. This approach to visualization allows simultaneous overview of predictions of binding to multiple HLA alleles.

### Example application: block conservation of HLA class I binders in influenza A H7N9 HA

**Figure 2:**
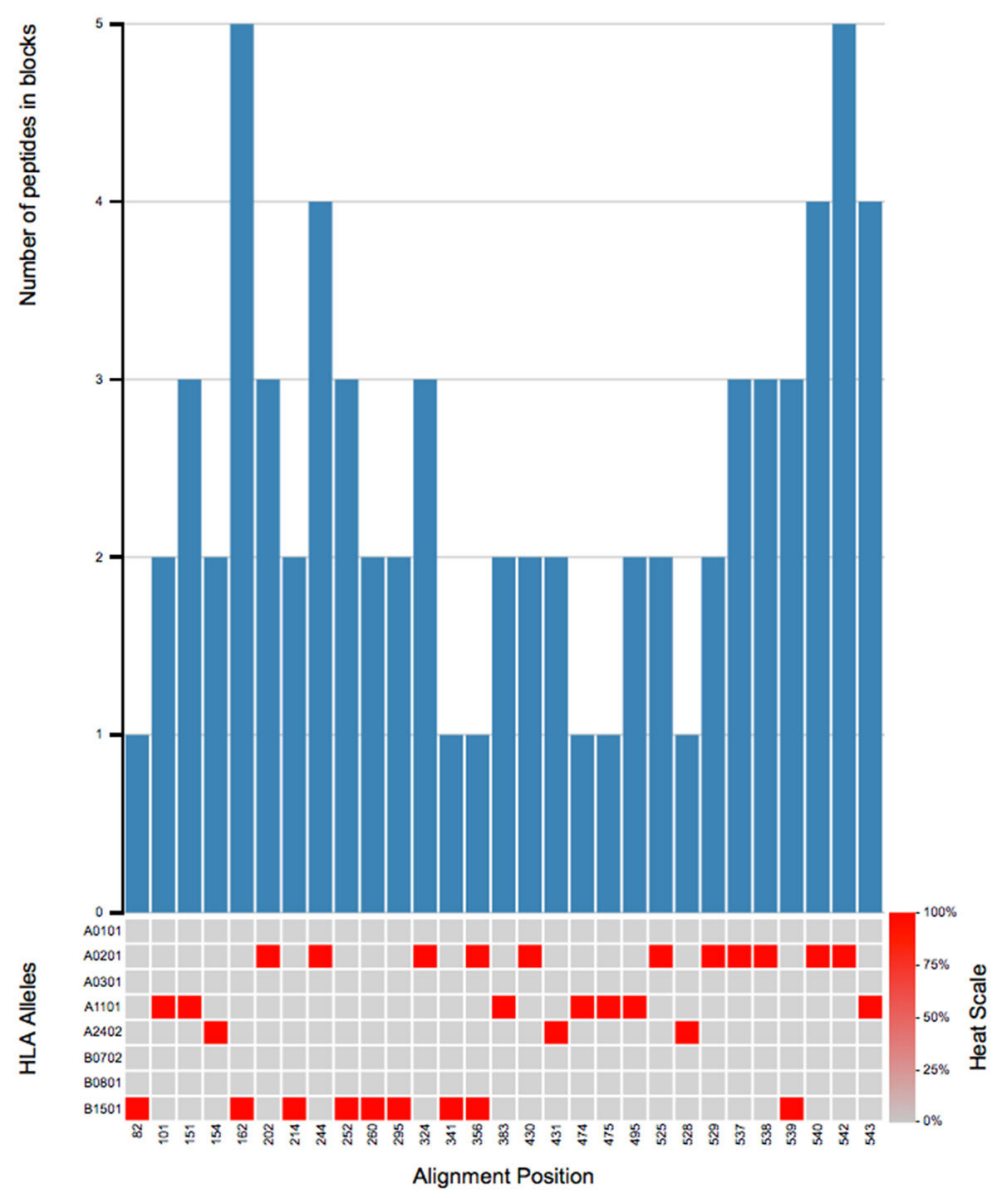
**Blocks of conserved HLA binding in a HA MSA**. Visualization of the block conservation analysis and HLA class I binding affinity predictions for 9mer blocks in which at least 99% of the peptides were predicted to bind at least one of the seven HLA I alleles used in this example. The bars show the number of peptides in a block (Y axis) at a given starting position in the MSA (X axis). The heat map below the bar shows the percentage of strains in the MSA predicted to bind to each of the HLA alleles predicted for in these examples. The color of each position in the heat map matrix ranges from blue (0% accumulated conservation by predicted binders in the block for the given allele) to red (at least 99% of the blocks are predicted to bind to the given allele with a minimum binding affinity of 500 nM).

We performed the block conservation analysis and prediction of epitopes from an MSA of the HA protein sequences. We found 29 conserved blocks in which all peptides were predicted to bind the same HLA (Figure 2). This compares favorably to traditional conservation analysis (positions where single predicted HLA binder is of at least 90% frequency) where less than 2% of these potentially immunogenic regions were captured.

**Figure 3:**
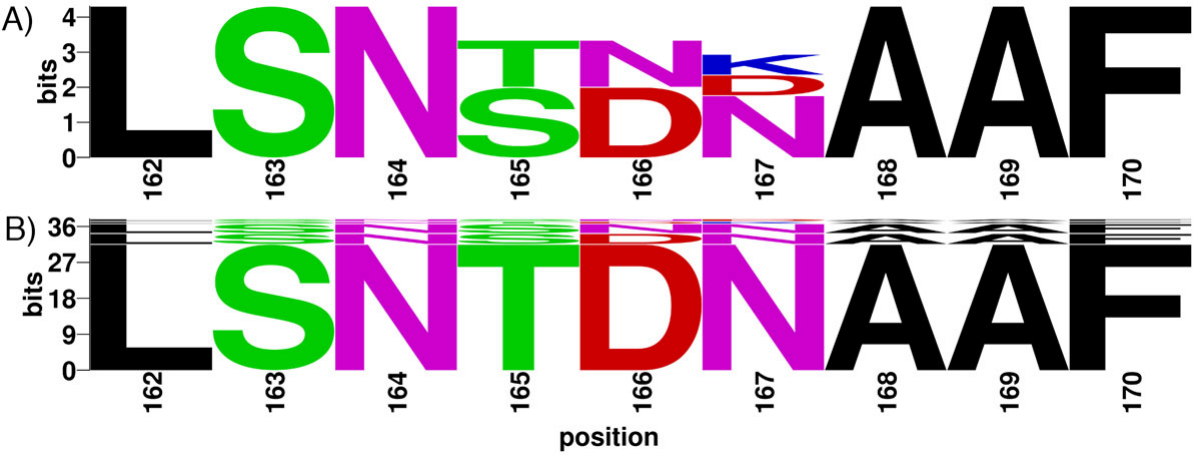
**Sequence conservation in influenza A HA block 162**. (A) Sequence logo and (B) BlockLogo visualizations of influenza A HA block 162.

To examine the characteristics of peptides in a given block, a BlockLogo [36] and a Sequence Logo [14] (generated using WebLogo [37]) were generated to visually represent conservation and variability of peptides and individual residues within block 162 of the influenza A HA protein represents an example of a region with conserved HLA binding. The intra-block diversity is shown in Figure 3, and a closer look at peptide HLA binding prediction in this block reveals that all peptides, despite not being highly conserved individually, are predicted to bind to B*15:01.

### Software implementation of method and visualization scheme

We developed a software implementation of the visualization scheme, which is freely available at http://met-hilab.cbs.dtu.dk/blockcons/. To generate a custom visualization, the user must submit an MSA of proteins of interest (pathogen or tumor proteins), and select analysis parameters as described below (also described in detail at http://met-hilab.cbs.dtu.dk/blockcons/html/instructions.php).

First, a block size between 8 and 25 amino acids (corresponding to the lengths of T-cell epitopes) can be selected. Then, a conservation threshold (the minimum accumulated frequency of peptides in a block, required for the block to be considered adequately covered) for each block can be selected. Most blocks will contain a large number of very low frequency variants, which can be filtered from the block if desired. For example, a peptide present only in a small fraction of examined viral proteins may be considered evolutionarily unstable (one or a few occurrences isolated in time and geographic location), and may be exempt from the analysis if desired. For this purpose, a conservation threshold of, for example, 99% can be chosen (i.e. filtering out peptides of less than 1% conservation). Similarly, this threshold acts as an immunological conservation threshold, i.e. the minimum accumulated frequency of predicted HLA binders in a block, in order for the block to be considered adequately conserved in terms of potential HLA binding.

To efficiently summarize the data, the size of the visualization can be reduced based on a user-defined threshold for displaying blocks. The HLA binding conservation threshold will filter the output by displaying only blocks in which the minimum accumulated frequency of HLA binders is above the threshold.

## Conclusions

The introduction of computational methodologies to vaccinology has enabled a significant step towards reverse vaccine design. The current paradigm for antigen selection is based on assembling a number of highly conserved peptides predicted to bind HLA, to cover diversity of pathogen and host with the smallest number of peptides. From a practical point of view, it would be cheaper and faster to experimentally validate fewer candidates, technically easier to include fewer peptides in vaccine designs, and the final epitope pool carries smaller risk of undesired immunodominance. However, from an evolutionary point of view, some viruses susceptible to treatment are likely to escape due to high mutation rates. Indeed, recent experimental efforts have shown that the TE of highly variable viruses tends to be rather low [20,38,39], so the inclusion of low-frequency epitopes in vaccines may decrease immune escape by these viruses.

We have developed a novel method and that integrates conservation analysis and prediction of immunogenic potential of peptides within viral antigens for fast identification of potential cytotoxic T lymphocyte (CTL) vaccine targets. The results of this analysis have provided an order of magnitude more potential targets as compared with traditional approaches. We addressed the problems of scale and integration of multiple results by designing a visual representation for rapid identification of potential polyvalent vaccine targets. The software implementation is freely available at http://met-hilab.cbs.dtu.dk/blockcons/.

## List of abbreviations used

CTL: Cytotoxic T lymphocyte; DENV: Dengue virus; HA: Hemagglutinin; HLA: Human leukocyte antigen; MSA: multiple sequence alignment; TE: Targeting efficiency.

## Competing interests

Part of this work is related to patent application US 20130064843 - Brusic V, Olsen LR, Reinherz EL, Zhang GL, Simon C. "Identification of conserved peptide blocks in homologous polypeptides".

## Authors' contributions

LRO and VB conceived of the method. LRO and CS conceived of, and performed the example application. LRO, CS, UJK, FOB, OW, and GL wrote the software and implemented the webserver. All authors participated in the writing of the manuscript.

## Supplementary Material

Additional File 1 **Figure S1**: **Visualization of conservation and binding predictions of all DENV blocks to all HLA alleles for which predictions are available**. The bars show the minimum number of peptides in a block (Y axis) at a given starting position in the MSA (X axis) required to fulfill the user defined coverage threshold, yx. The heat map below the bar show the percentage of peptides in the block predicted to bind to each of the HLA alleles predicted for in these examples. The color of each position in the heat map matrix ranges from blue (0% accumulated conservation by predicted binders in the block for the given allele) to red (blocks predicted to bind to the given allele with a minimum binding affinity of 500 nM represents 99% conservation in the block). Alleles have been clustered to reflect similarity in binding properties. Results of clustering are summarized to the right of the heatmap. Click here for file

## Declarations

Publication charges for this article have been funded by the Novo Nordisk Foundation.

This article has been published as part of *BMC Medical Genomics *Volume 8 Supplement 4, 2015: Joint 26th Genome Informatics Workshop and 14th International Conference on Bioinformatics: Medical genomics. The full contents of the supplement are available online at http://www.biomedcentral.com/bmcmedgenomics/supplements/8/S4.
