# Supplementary material for: A computational method for identification of vaccine targets from protein regions of conserved human leukocyte antigen binding
Source: BMC Med Genomics. 2015 Dec 9;8(Suppl 4):S1. doi: 10.1186/1755-8794-8-S4-S1 (PMC4682376; doi:10.1186/1755-8794-8-S4-S1)
Supplement: Additional File 1 — Figure S1: Visualization of conservation and binding predictions of all DENV blocks to all HLA alleles for which predictions are available. The bars show the minimum number of peptides in a block (Y axis) at a given starting position in the MSA (X axis) required to fulfill the user defined coverage threshold, yx. The heat map below the bar show the percentage of peptides in the block predicted to bind to each of the HLA alleles predicted for in these examples. The color of each position in the heat map matrix ranges from blue (0% accumulated conservation by predicted binders in the block for the given allele) to red (blocks predicted to bind to the given allele with a minimum binding affinity of 500 nM represents 99% conservation in the block). Alleles have been clustered to reflect similarity in binding properties. Results of clustering are summarized to the right of the heatmap. [file 1755-8794-8-S4-S1-S1.pdf]

Figure S1: Visualization of conservation and binding predictions of all DENV blocks to all HLA alleles for which predictions are available.

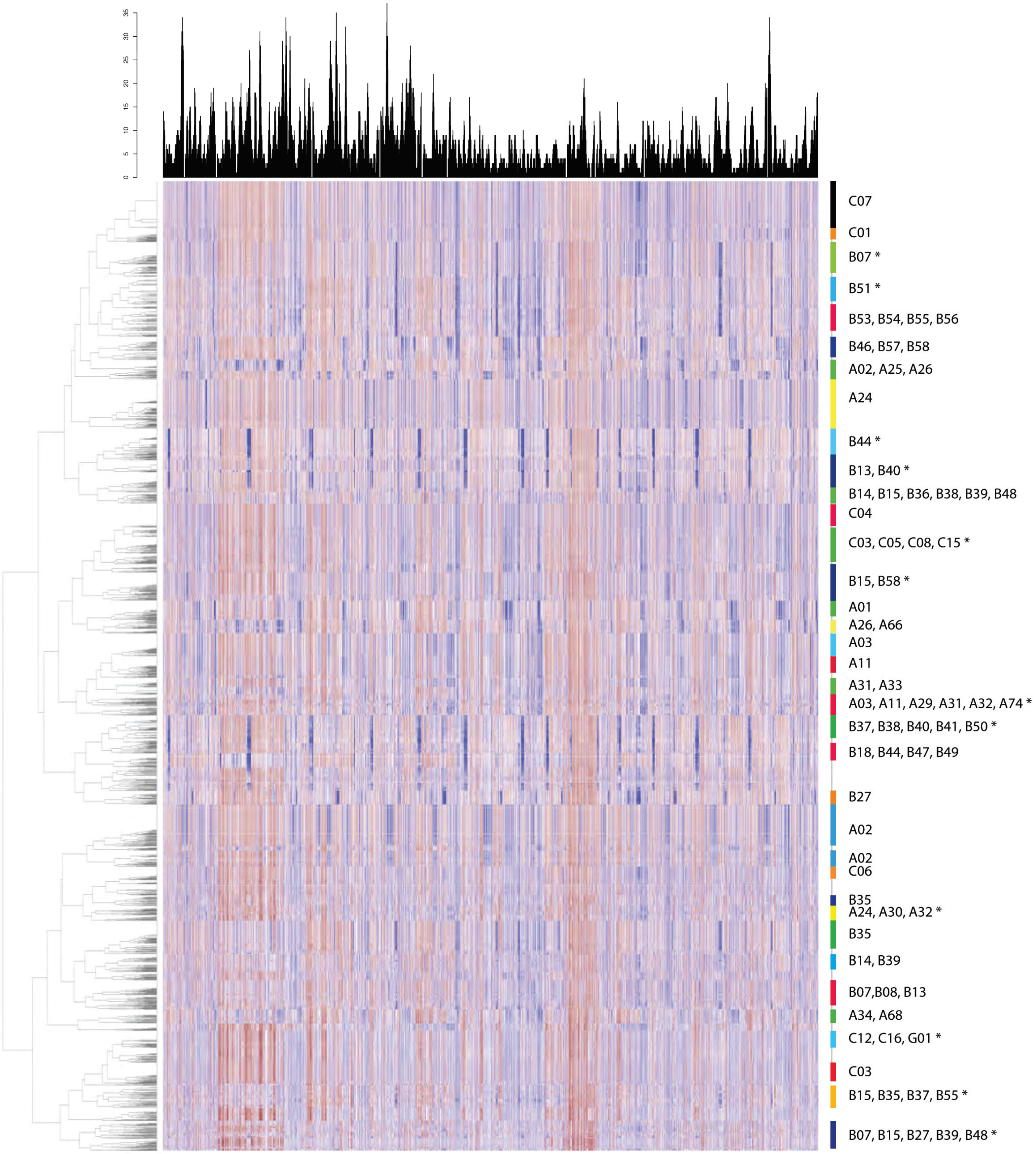

The bars show the minimum number of peptides in a block (Y axis) at a given starting position in the MSA (X axis) required to fulfill the user defined coverage threshold, yx. The heat map below the bar show the percentage of peptides in the block predicted to bind to each of the HLA alleles predicted for in these examples. The color of each position in the heat map matrix ranges from blue (0% accumulated conservation by predicted binders in the block for the given allele) to red (blocks predicted to bind to the given allele with a minimum binding affinity of 500 nM represents 99% conservation in the block). Alleles have been clustered to reflect similarity in binding properties. Results of clustering are summarized to the right of the heatmap.
